# Uterine Artery Embolization in Women with Symptomatic Cervical Leiomyomata: Efficacy and Safety

**DOI:** 10.1007/s00270-018-2081-2

**Published:** 2018-10-04

**Authors:** Annefleur M. de Bruijn, Sven-Ole J. H. Adriaansens, Marieke Smink, Alexander Venmans, Wouter J. K. Hehenkamp, Albert J. Smeets, Anthony Lopez, Judith A. F. Huirne, Paul N. M. Lohle

**Affiliations:** 1 Department of Gynecology, VU Medical Center, De Boelelaan 1117, 1007 MB Amsterdam, The Netherlands; 2 Department of Radiology, The Royal Surrey County Hospital, Guildford, England, UK; 3 Department of Radiology, Elisabeth Tweesteden ziekenhuis, Tilburg, The Netherlands; 4 Department of Gynecology, Elisabeth Tweesteden Ziekenhuis, Tilburg, The Netherlands

**Keywords:** Cervical leiomyomata, Uterine artery embolization, Health-related quality of life (HRQOL)

## Abstract

**Purpose:**

To perform an evaluation on safety and efficacy of uterine artery embolization (UAE) in the patients with symptomatic cervical leiomyomata.

**Methods:**

Patients with symptomatic cervical leiomyomata who underwent UAE in one specialized hospital were retrospectively analyzed, both clinically and with MR imaging. The 3-month outcomes were assessed with MR imaging and a validated questionnaire. Long-term follow-up was assessed by direct contact or file review. To determine the efficacy of UAE for cervical leiomyomata, the primary objective was to assess the clinical outcome with the UFS-QOL questionnaire, containing the health-related quality of life (HRQOL) and symptom severity score (SSS). To assess safety, the secondary objective included leiomyomata volume reduction, the infarction/complication rate and secondary interventions were needed.

**Results:**

Between 2006 and 2017, eight of 1180 patients underwent UAE and were eligible for inclusion. All embolizations were technically successful (*n* = 8). At 3 months, all patients showed cervical leiomyomata volume reduction with a median reduction of 41.5% (38.8 cm^3^) compared to baseline (*p* = 0.012). No complications occurred. At a median follow-up of 3 months (range 1–7, *n* = 7), the HRQOL and SSS improved with a median difference of 13 points (range − 5 to 60, *p* = 0.063) and − 13 points (range − 79 to 3, *p* = 0.046), respectively. Long-term follow-up showed two secondary interventions (median of 43.5 months). Six patients reported no symptom recurrence.

**Conclusion:**

UAE in women with symptomatic cervical leiomyomata is effective and safe with significant improvement in symptoms and quality of life. UAE is a valuable option for women seeking a non-surgical solution.

**Electronic supplementary material:**

The online version of this article (10.1007/s00270-018-2081-2) contains supplementary material, which is available to authorized users.

## Introduction

Uterine leiomyomata are common benign tumors originating from neoplastic transformation of smooth muscle cells in the uterine wall [1]. Approximately 20–40% of women are affected in their reproductive age [2]. Uterine leiomyomata located in the cervix are rare, ranging from 0.9 to 8% of all uteri containing leiomyomata [3–5]. Symptoms associated with cervical leiomyomata are abnormal bleeding, pain (dysmenorrhea) and bulk-related symptoms [6]. Surgical treatment of cervical leiomyomata is difficult due to its location. Poor access to the operating field, suturing difficulty, poor cervical flexibility, increased blood loss and close neighboring organs (bladder, ureter and rectum) could hamper the procedure [7, 8]. UAE is an established valuable treatment alternative to surgery in the treatment of uterine fibroids located in the body or fundus of the uterus as reported in multiple randomized controlled trials [9–14]. Identification of the cervical vessels is complex and often differs in shape, size and location. Literature describes that the branches running toward the cervix are significantly variable in patients and shows that the vascular supply of the cervix seems to come from several vessels; however, these findings are based on postmortem studies [15–18]. Therefore, the localization of the afferent artery to the cervical perifibroid plexus is challenging. The aim of our study was to evaluate the efficacy and safety of UAE in the treatment of symptomatic cervical leiomyomata. This was achieved by using the validated standard UFS-QOL questionnaire including the health-related quality of life (HRQOL) and symptom severity score (SSS) [19, 20]. In addition imaging outcomes, complications and secondary interventions were evaluated.

## Materials and Methods

### Study Design

This retrospective study evaluated all patients that underwent UAE for cervical leiomyomata from 2006 until 2017 in a single specialized hospital in the Netherlands. To determine the efficacy of UAE for cervical leiomyomata, the primary objective was to assess the clinical outcome using the validated standardized questionnaire Uterine Fibroid Symptom and Health-related Quality of Life (UFS-QOL) at baseline and at 3 months after UAE, containing the health-related quality of life (HRQOL) and symptom severity score (SSS). To assess safety, the secondary objective included cervical leiomyomata volume reduction, the infarction/complication rate and secondary interventions needed. Inclusion criteria were (1) patients with leiomyomata-related complaints, i.e., abnormal uterine bleeding, pain (dysmenorrhea) and/or bulk-related symptoms, (2) MR imaging which confirmed the presence of a cervical leiomyomata with or without other uterine body leiomyomata, (3) leiomyomata treated with UAE and (4) the availability of 3 months post-embolization MR images. Exclusion criteria were (1) UAE during pregnancy and (2) UAE for postpartum hemorrhage. The local ethics committee approved this study. All patients gave their informed consent for retrospective review with a waiver for patients who were unable to be located.

### Study Measures

#### Magnetic Resonance Imaging (MRI) at Baseline and Follow-Up (Short Term and Long Term)

All patients included underwent T1, T2 and T1 contrast-enhanced (gadolinium) MR imaging at baseline and at 3 months following UAE. The diagnosis of a cervical leiomyomata was determined with MR imaging with the criterion of leiomyomata location at the cervix (intramural/submucous/subserous) or attachment (pedunculated) to the cervix. Fibroid volume was calculated using the ellipsoid formula: length × width × height × 0.5233. Two radiologists assessed after UAE the infarction rate by using the same volumetric measurement as mentioned above [21]. Insufficient infarction was determined at a cutoff point < 80% as described earlier by Smeets et al. [22].

#### Angiographic Procedure

**Fig. 1:**
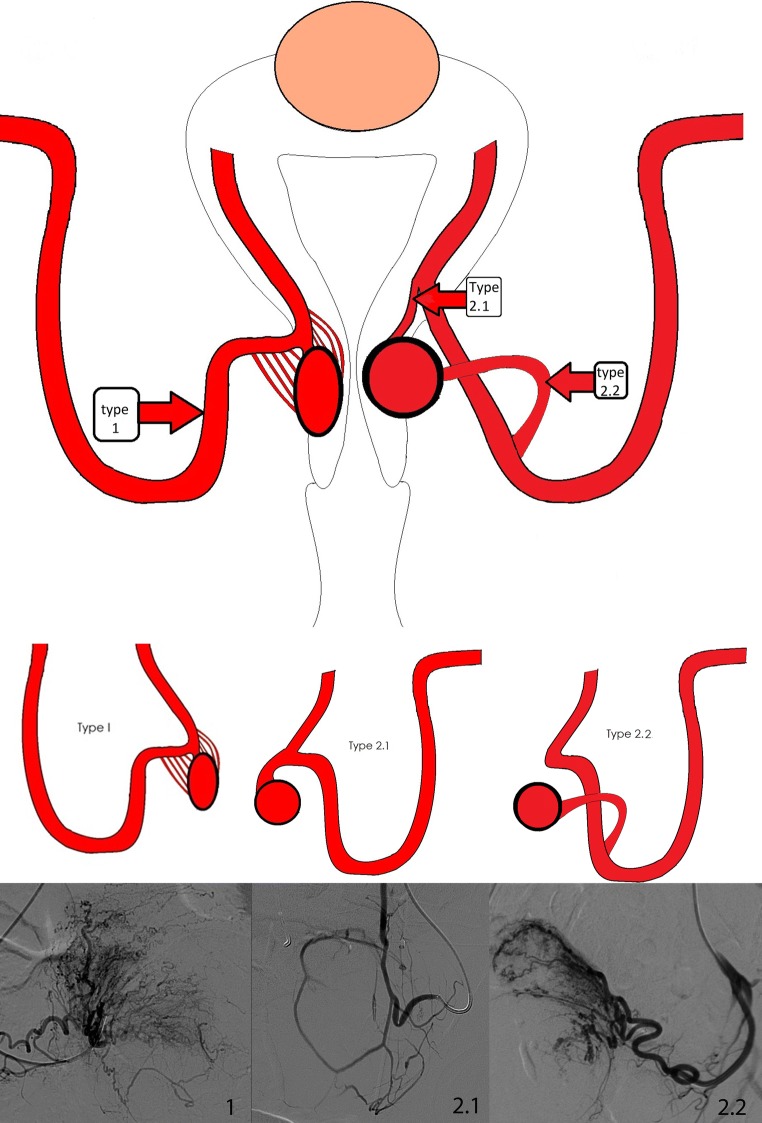
Overview of afferent branching arteries to cervical leiomyomata as identified during UAE. *Note* Boxes type 1, 2.1 and 2.2 are the locations of the catheters as used per subtype. Subtype 1 with a plexus of feeding branches to the cervical leiomyomata. Subtype 2.1 with a proximal single branch from uterine artery to the cervical leiomyomata. Subtype 2.2 with a distal single branch from uterine artery to the cervical leiomyomata. Type 2 cases were, when possible, super-selectively catheterized

UAE was performed according to local protocol and professional standards and reported as described by Goodwin et al. [23]. All patients received a drip infusion at the wrist and an intravenous patient-controlled analgesia pump for pain management. All UAE consisted of bilateral access in the common femoral arteries with placement of the 4Fr sheath and selective placement of a C2 catheter (Tempo Aqua C2, Cordis Corporation, Miami, USA) in both uterine arteries. Digital subtraction angiography (DSA) displayed the cervical leiomyomata vascularity. According to Kim et al. [24], the cervical leiomyomatas were classified based on vascular aspects during DSA: Grade I poor/minimal vascularity, without enhancement of the tumor; Grade II moderate vascularity, with limited enhancement in small vessels intratumorally; and Grade III pronounced staining of the leiomyomata, with large tortuous vessels. In addition to these intratumoral vascularity patterns, an overview is made of two types of afferent arteries identified during DSA (Fig. 1). Due to a lack of available information (sometimes lack of detailed DSA images of the previous procedures) and overprojection of Grade III tortuous vessels, not all the patients could be classified. However, the radiologists performing the embolization procedures described the different types of afferent arteries as seen during the procedures in their reports. Ideally, a catheterization technique was applied when possible with super-selective placement of the (micro-)catheter tip into the uterine artery side branch, i.e., a separate cervical artery, in order to secure optimal embolization of the cervical leiomyomata. Initial treatment was focused on super-selective UAE of the cervical fibroid followed by standard UAE of the remaining uterine fibroids when present. Figure 1 displays an overview of the different afferent arteries and the location of the catheter during UAE. Described in this cohort is the dominant side of the afferent artery. Type 1 is an often seen entity, wherein not only one afferent artery can be identified due to overprojection of an arterial plexus. Potentially, there is no solitary afferent artery, but only a plexus feeding the cervical leiomyomata. In Type 1 cases with an arterial plexus, the catheter tip was positioned at point A. Type 2 was identified in 3 cases, defined as a cervical leiomyomata with a single uterine artery branch, i.e., a solitary feeding cervical artery. In Type 2 cases, when possible, super-selective positioning of the catheter tip was applied into the afferent cervical artery. Type 2.1 is a solitary side branch originating proximal from the ascending segment of the uterine artery. Type 2.2 is a distal solitary side branch of the uterine artery.

The embolization agents used were precisely calibrated microspheres, built of a hydrogel core and coated with a polymer Polyzene^®^-F (Embozene™, Boston Scientific, Amsterdam, The Netherlands) ranging in size from 500 to 1200 µm and additionally 700 µm non-spherical polyvinyl alcohol (PVA) particles in one patient (Contour™, Boston Scientific, Amsterdam, The Netherlands). The angiographic embolization endpoint was at complete stasis. All patients received a periprocedural intravenous patient-controlled analgesia pump, for adequate pain treatment. Complications were recorded.

#### Clinical Assessment at Baseline and Follow-Up (Short Term and Long Term)

Patients completed the standardized questionnaire, Uterine Fibroid Symptom and Health-related Quality of Life (UFS-QOL) at baseline and at 3 months after UAE [20, 25]. Adverse events were recorded. Long-term clinical results were also obtained through a telephone-administered questionnaire and reviewing patients’ files. The UFS-QOL rates the SSS on a scale of 0–100 and the HRQOL on a scale of 0–100 in seven different domains: (1) concern, (2) activities, (3) energy/mood, (4) control, (5) self-consciousness, (6) sexual function and (7) HRQOL total score [20]. A lower SSS means improvement in symptoms. A higher HRQOL score indicates better quality of life. The duration of hospital stay was recorded.

### Statistical Analysis

The Wilcoxon signed-rank test for paired samples was used to compare the cervical leiomyomata volumes, the UFS-QOL including HRQOL and symptoms severity scores (SSS) during follow-up and at baseline. *P* values < 0.05 were considered statistically significant. SPSS (IBM SPSS Statistics, version 22) was used for statistical analysis.

## Results

From a total of 1180 patients who underwent UAE during 2006 until 2017, 12 patients with proven cervical leiomyomata on MRI underwent UAE. Four of these patients were excluded due to pregnancy (*n* = 1), postpartum (*n* = 1), UAE of an acute bleeding leiomyomata expulsion as preparation for surgical removal (*n* = 1) and absence of a follow-up MRI (*n* = 1). The median age of all patients (*n* = 8) at baseline was 37.0 years, ranging from 33 to 47 years. Three patients showed concurrent non-cervical fibroid disease (Table 1, patients 6, 7 and 8). Table 1 lists baseline characteristics of these patients with symptomatic cervical leiomyomata (demographics, previous treatment, symptoms and concurrent leiomyomata) who underwent UAE (procedural characteristics). All eight UAEs were technically successful without complications. All patients were discharged from the hospital the next day, except one patient who needed one extra night due to persistent pain. After 3 months, one patient reported transient amenorrhea.

**Table 1:** Patient demographics and procedural characteristics

Patient demographics, previous treatment, symptoms				Procedural characteristics		
Patient	Age	Previous treatment	Main symptoms	Catheter	UAE material	Hospital stay
1	33	Hormonal (oral)	BRS + WTC	4Fr sheath + C2	Microspheres 900–1100 µm	1
2	37	NR	AUB + WTC	4Fr sheath + C2	Super-selective. Microspheres 500, 700, 900 µm (only left side)	1
3	33	Tranexamic acid, ulipristal. Myomectomy 2007 and 2015	BRS + WTC	4Fr sheath + C2	Microspheres 700, 900, 1300 µm and 700 µm PVA	1
4	45	NR	BRS	4Fr sheath + C2	Microspheres 700–900 µm	1
5	38	Hormonal (oral)	P	4Fr sheath + C2	Microspheres 500, 900 µm	1
6+	47	NR	AUB + BRS	4Fr sheath +C2	Microspheres 700, 900, 1200	1
7+	42	No	AUB	4Fr sheath + C2	Microspheres 700–900 µm and 900–1200 µm	3
8+	33	Hormonal (oral)	P + AUB	4Fr sheath + C2	Microspheres 700–900 µm	1
Median (range)	37.5 (33–47)					1.0 (1–3)

*NR* not reported, *AUB* abnormal uterine bleeding, *BRS* bulk-related symptoms, *WTC* wish to conceive, *P* intermittent pain not related to the menstrual cycle

+ With concurrent non-cervical leiomyomata disease

### Imaging Results

**Fig. 2:**
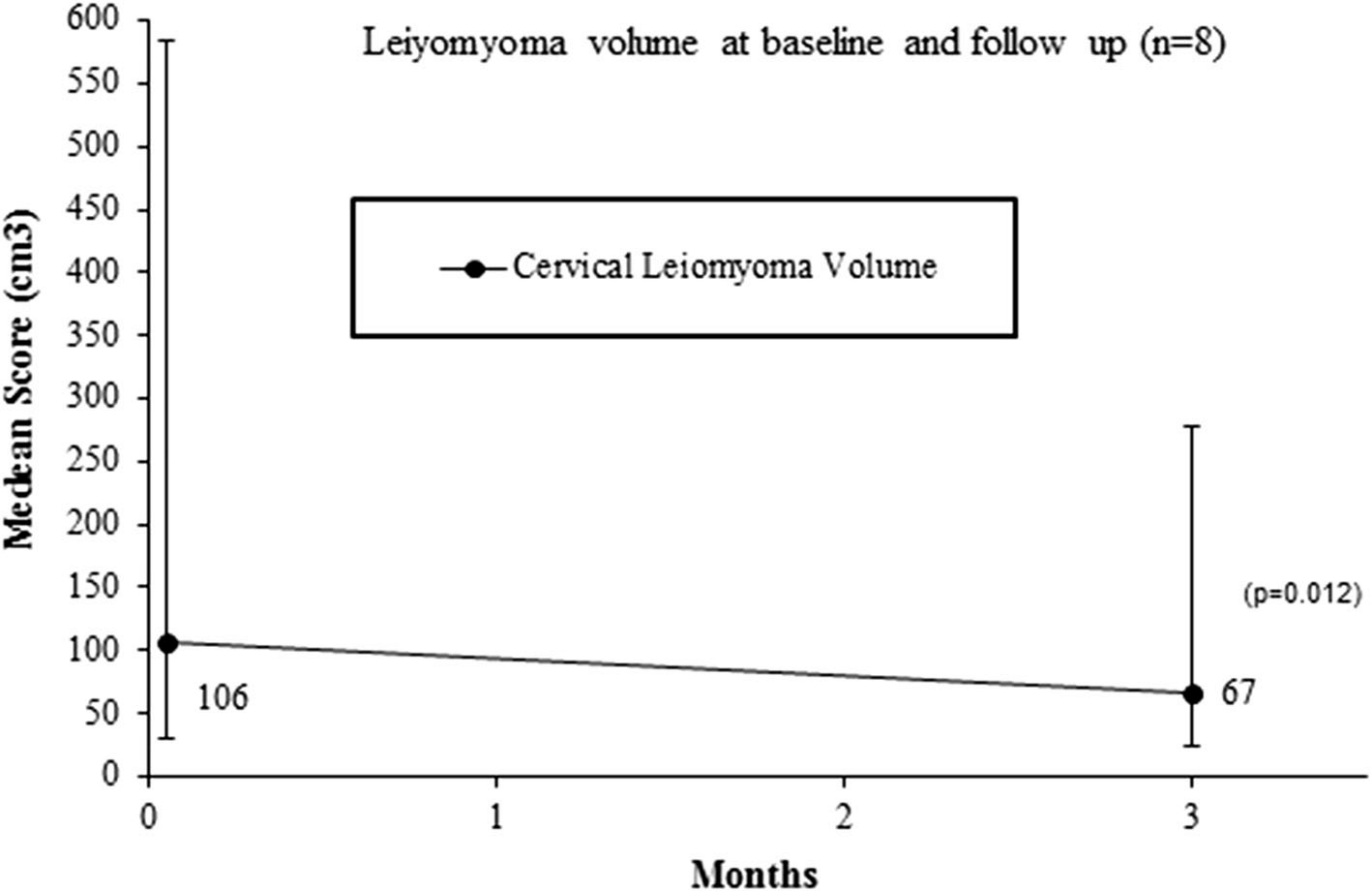
Median cervical leiomyomata volume reduction (cm^3^) until 3 months of follow-up compared to baseline

All patients demonstrated volume reduction compared to baseline at 3 months after UAE (Table 2). Figure 2 displays a median leiomyomata volume reduction of 41.5% (38.8 cm^3^) at 3 months compared to baseline (*p* = 0.012). Five out of eight patients (62.4%) displayed ≥ 80% infarction of the cervical leiomyomata. The grade pattern of these patients is displayed in Table 2. Three patients (no. 1, 7 and 8) demonstrated 50, 40 and 60% infarction of the cervical leiomyomata, respectively (Table 2). All of these patients had a Grade III leiomyomata.

**Table 2:** Imaging characteristics at baseline and follow-up

Baseline					3-Month follow-up					
Patient	Grade	Size (cm)	Volume (cm^3^)	Body fibroid Volume (cm^3^)	Size (cm)	Volume (cm^3^)	Volume decrease	Infarction rate	Body fibroid volume reduction	*Body fibroid* *Infarction rate*
*Patients with solitary cervical leiomyomata*										
1	3	8.4 × 8.6 × 7.8	294.86	–	6.4 × 6.7 × 5.5	123.41	58.1%	50%	NA	NA
2	3	5.9 × 6.1 × 5.6	105.47	–	4.4 × 4.9 × 5.0	56.41	46.5%	95%	NA	NA
3	3	7 × 8×12.8	375.1	–	5.7 × 8.1 × 11.5	277.85	25.9%	80%	NA	NA
4	3	6.0 × 5.6 × 5.1	89.67	–	4.9 × 4.1 × 3.8	39.95	55.5%	90%	NA	NA
5	1	5.8 × 4.3 × 4.1	53.50	–	4.4 × 4.1 × 3.6	33.98	36.5%	100%	NA	NA
Median (range)			105.5 (53.5–375.1)			56.4 (34.0–277.9)	46.5% (25.9–58.1)	90% (50–100)		
*Patients with concurrent non*-*cervical fibroid disease*										
6	2	7.2 × 5.2 × 5.4	105.80	837.71	5.0 × 4.4 × 6.7	77.13	27.1%	100%	21.6%	95%
7#	3	11.5 × 9.7 × 10.0	583.74	1: 9.18 2: 14.13 3: 3.3	7.5 × 8.0 × 6.9	216.65	62.9%	40%	1: 73.4% 2: 37.4% 3: 48.5%	1:100% 2:100% 3:100%
8#	3	3.4 × 3.6 × 4.6	29,46	1:6.88 2: 59.77	3.4 × 3.2 × 4.1	23.34	20.8%	60%	1: 34.0% 2: 26.1%	1: 90% 2: 0%
Median (range)			105.8 (29.5–583.7)			77.1 (23.3–216.7)	27.1% (20.8–62.9)	60% (40–100)		
*Total of patients*										
Total median (range)			105.6 (29.46–583.8)			66.8 (23.3–277.9)	41.5% (20.8–62.9)		85% (40–100)	

*NA* not applicable

# Patient underwent secondary intervention

**Fig. 3:**
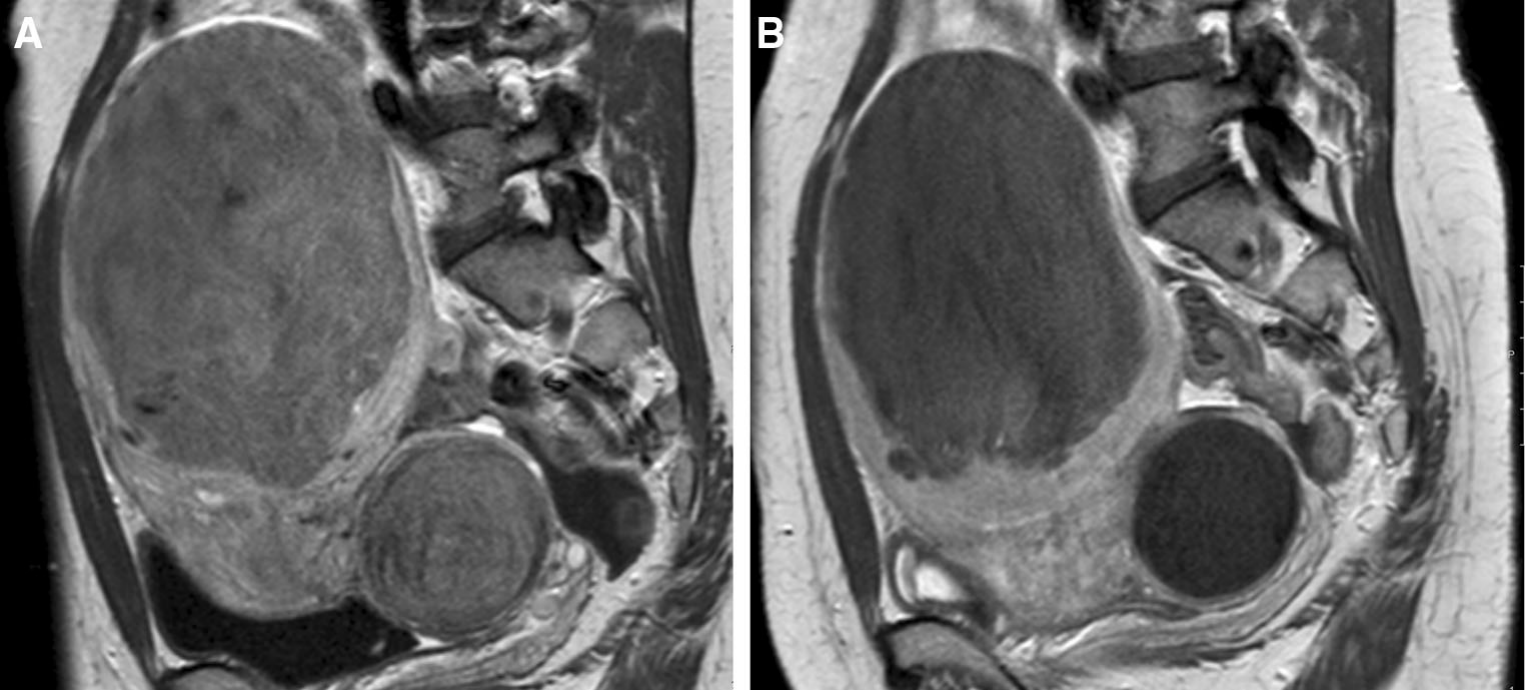
MRI (*SAG T1* − *TSE* − *HR contrast* +) imaging of cervical leiomyomatas in combination with uterine body fibroids. *Note* Patient no. 6, MR images (sagittal T1 − TSE − HR + contrast) of leiomyomata in the uterine body and cervix. **A** Both the cervical leiomyomata (106 cm^3^) and the uterine body leiomyomata demonstrated full enhancement prior to UAE. **B** Follow-up imaging 3 months after UAE with 5% enhancement of the leiomyomata in the uterine body and no enhancement of the cervical leiomyomata, as a result of complete infarction after bilateral UAE (2cc700 µm, 4cc900 µm and 4cc1200 µm microspheres, Embozene™). The cervical leiomyomata volume 3 months after UAE was 77 cm^3^ with a volume reduction of 27%

**Fig. 4:**
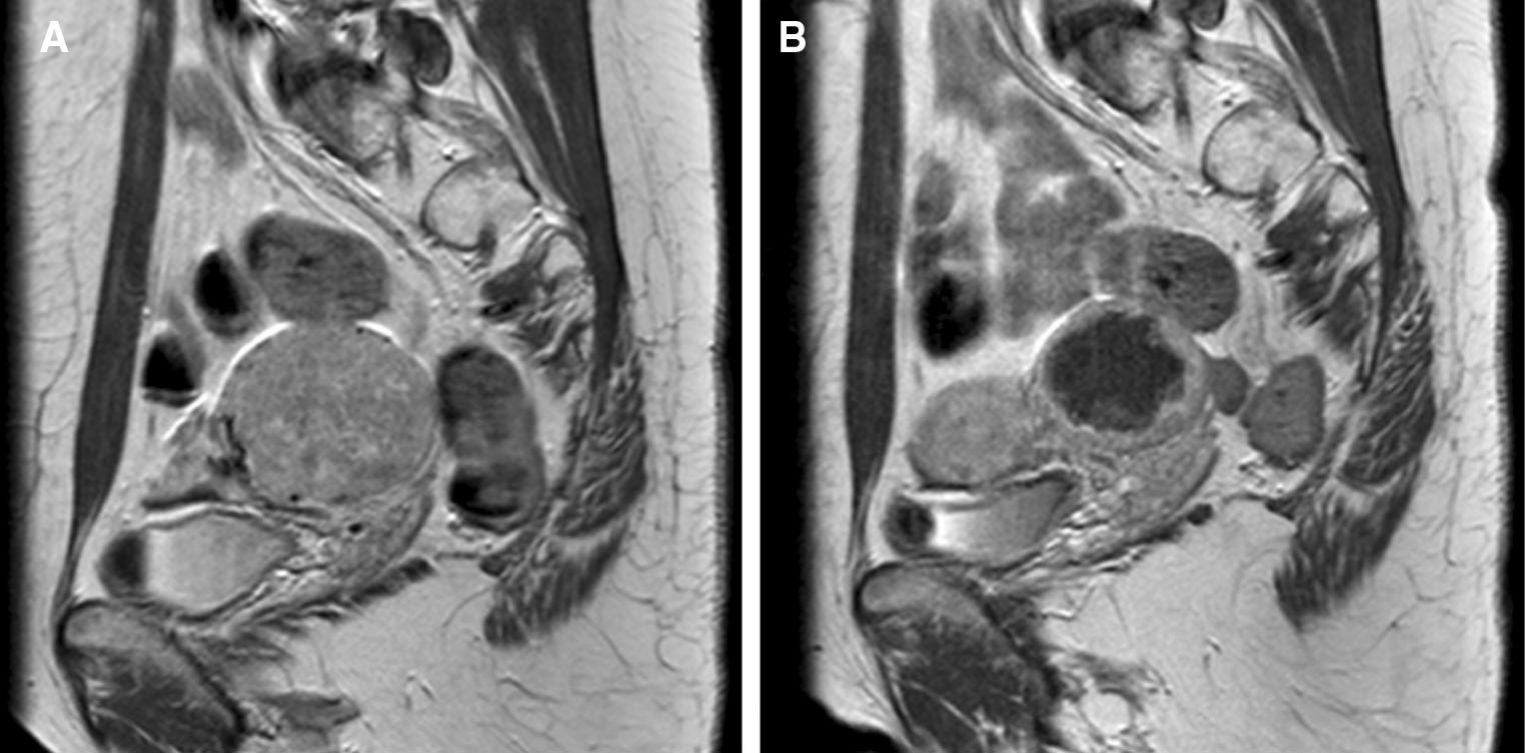
MRI (*SAG T1* − *TSE* − *HR contrast* +) imaging of a solitary cervical leiomyomata. *Note* Patient no. 2, MR images (sagittal T1 − TSE − HR + contrast) of a single cervical leiomyomata in a 37-year-old patient with heavy menstrual blood loss and a wish to conceive. Cervix with a broad-based leiomyomata on the left. **A** The cervical leiomyomata (106 cm^3^) with full enhancement prior to UAE. **B** MR imaging 3 months after UAE showed 95% infarction with an irregular enhancing rim. Unilateral left-side UAE with 2cc500, 2cc700 and 3cc900 µm microspheres Embozene™; 47% volume reduction (56.4 cm^3^)

Three out of eight patients (no. 6, 7 and 8) demonstrated concurrent non-cervical leiomyomata disease, and two of them (no. 7 and 8) received additional treatment. These two patients showed a cervical leiomyomata infarction rate of 40% and 60%. In patient no. 8, a concurrent anterior wall leiomyomata showed no infarction after the first UAE. Follow-up MRI demonstrated an unchanged uterine body leiomyomata and progressive enhancement of the cervical leiomyomata overtime. Results of the (technically successful) secondary UAE showed no additional infarction rate on the follow-up MRI. Patient no. 7 showed an infarction rate of 40% (cervical leiomyomata) after the initial UAE, with a volume reduction of nearly 63% (216.7 cm^3^). Due to persisting gynecological symptoms with complaints of mass effect, hysterectomy was eventually carried out. Figures 3 and 4 display the image changes in terms of aspect, volumes and contrast enhancement of cervical leiomyomata after UAE in patient no. 2 and 6.

### Clinical Results UFS-QOL: Short Term

**Fig. 5:**
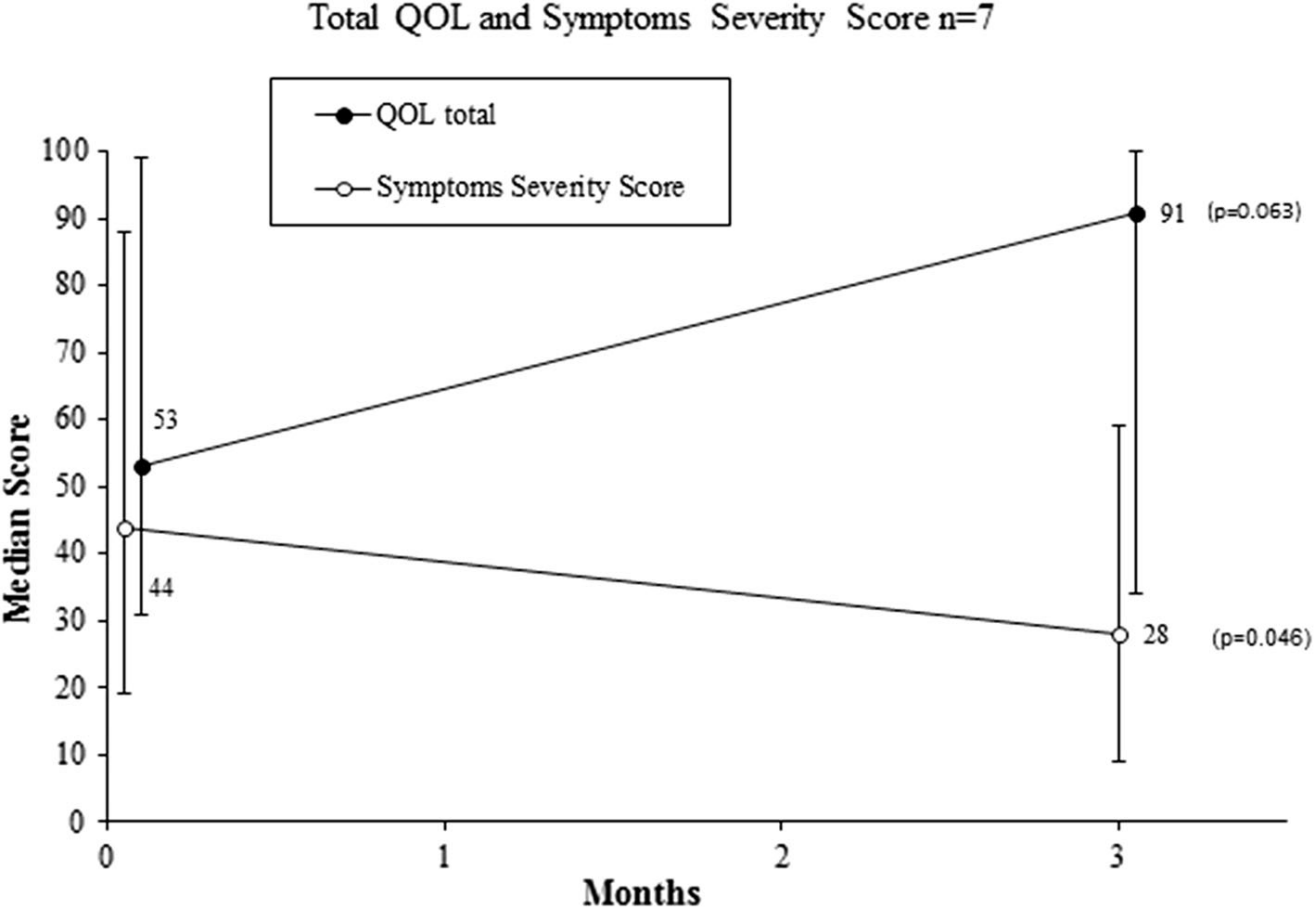
Total quality of life and symptoms severity scores at baseline and 3 months of follow-up

Seven of eight included women filled out the UFS-QOL at a median follow-up of 3 months (range 1–7). Figure 5 depicts the median HRQOL and SSS (both on a total scale of 100 points) at baseline and follow-up. The total HRQOL score showed a median, nonsignificant increase of 13 points (range − 5 to 60; *p* = 0.063). The SSS demonstrated a statistically significant improvement compared to baseline and decreased with a median score of − 13 points (range − 79 to 3; *p* = 0.046). Appendix 1 in electronic supplementary material displays the median score of the separate domains. It shows an increase in HRQOL scores in every domain; however, only sub-domains concern (*p* = 0.043), control (*p* = 0.046) and self-consciousness (*p* = 0.042) reached statistical significance.

### Clinical Outcomes: Long Term

Long-term clinical follow-up with a median of 43.5 months (range 6–127, *n* = 8) outlined the two secondary treatment cases. Patient no. 8 with concurrent non-cervical uterine leiomyomata disease received a second UAE at 15 months due to recurrent pain after 12 months. At 17 months following the second UAE, she returned again because of pain in the left lower abdomen. At 107 months after the second UAE, no additional treatment was necessary and reported symptoms to be manageable. Patient no. 7 needed a hysterectomy at 72 months after UAE due to cervical leiomyomata growth and clinical symptom recurrence. Patient no. 5 with reported worsening of HRQOL and limited improvement in symptoms in short term became asymptomatic on long-term follow-up (45 months). The remaining patients (*n* = 6, 75%) reported no additional treatments needed nor symptom recurrence at a median follow-up of 38.0 months (range 6–125). No adverse events were reported.

## Discussion

### Summary of Clinical Findings

This retrospective study demonstrated the efficacy and safety of UAE in women with symptomatic cervical leiomyomata, based on clinical outcomes with HRQOL, symptom severity scoring (SSS) and MR imaging. Significant reduction in the cervical leiomyomata volume was calculated related to a statistically significant decrease in the SSS at 3 months after UAE. Short-term improvement in symptom severity and HRQOL seems to be largest in the women with concurrent non-cervical leiomyomata disease. However, long-term outcomes displayed that this effect was not maintained. UAE infarction rates were ranging between 40 and 100%. Six out of eight patients underwent a successful cervical leiomyomata UAE treatment. All of these patients with the exception of one showed a leiomyomata infarction rate of more than 80%. Two of three patients with concurrent non-cervical leiomyomata disease received additional therapy because of recurrent or persisting symptoms. No complications occurred.

### Interpretations of Outcomes

Patients with a solitary cervical leiomyomata showed lower median improvement in HRQOL and symptom severity at short-term follow-up compared to women with concomitant uterine fibroids. This could be explained by the limited number of patients in this study and the effect of two patients with solitary cervical leiomyomata demonstrating low UFS-QOL scores, which may be explained by a lower infarction rate and accompanying non-cervical leiomyomata disease. As suggested by Aryani et al. [26], a potential cause of insufficient cervical leiomyomata infarction could be preexistent collaterals potentially also feeding the cervical leiomyomata. This was unfortunately not verifiable, because no specific DSA examination of the internal iliac arteries with their anterior divisions to the pelvis with potential collaterals was performed during the UAE procedures. Another cause of incomplete infarction might be the hypervascularity of the body/fundal leiomyomata, in which case the embolic agent might migrate to the uterus instead.

### Strengths and Limitations

This is the second retrospective study published about UAE in women with symptomatic cervical leiomyomata, Kim et al.’s study being the first [24]. Our study retrospectively examined the clinical outcome before and after UAE obtained by validated questionnaires, which were prospectively collected. All available imaging was evaluated by two radiologists, making outcomes more reliable. Only eight patients were included in this study, resulting in a limited sample size. Therefore, although the UAE results in symptomatic cervical leiomyomata favor this option as a treatment for women seeking uterine-sparing surgery, the limited sample size does not allow us to draw too strong conclusions. Larger high-quality trials should be conducted to confirm our results. However, this condition is rare; thus, reports like these remain important when counseling these patients.

### Comparison with Other Studies

We are aware of two reports [24, 27] on UAE in the treatment of cervical leiomyomata. The first report compared effectiveness of UAE of cervical leiomyomata versus uterine fundal/body leiomyomata with imaging and stated that “the results of UAE were disappointing, indicating a need for caution in selecting and counseling patients for this treatment” [24]. At 3 months, they reported a total infarction in only 2/10 (20%) patients and zero infarction in 2/10 (20%) patients, with symptom improvement in 4/10 (40%) patients. They did not use a standardized validated questionnaire. Five out of nine patients showed a Grade I vascularity pattern, two Grade II and two Grade III vascularity pattern. Our cohort consists of eight patients with six Grade III vascularity patterns, one Grade II and one Grade I vascularity pattern.

Our study results demonstrated a higher treatment success rate. At 3 months, the symptom severity score (SSS) and HRQOL score improved in 7/7 patients (100%) and 5/7 patients (71%), respectively. Long-term satisfaction without additional therapy was achieved in 6/8 patients (75%). The difference in success rate may be explained by the leiomyomata vascularity (Grade III), a different UAE agent and the relatively larger size, and therefore, the relatively increased vascularization which might presumably lead to increased infarction compared to infarction rates of smaller fibroids with less vascularization and/or the described catheterization technique.

The second publication concerns the world’s first reported UAE during pregnancy [27]. We hypothesized that the pregnancy itself would be an influencing factor on the cervical leiomyomata growth, which is why we excluded pregnant or postpartum patients in this study [28].

## Conclusion

UAE in women with symptomatic cervical leiomyomata seems to be effective and safe with significant improvement in symptoms and quality of life. UAE is a valuable option for women seeking a non-surgical solution.

## Electronic supplementary material

Below is the link to the electronic supplementary material.

Appendix 1 Median quality of life subdomain scores at baseline and 3-month follow-up (TIFF 10035 kb)
