# Supplementary figures and images for: Uterine Artery Embolization in Women with Symptomatic Cervical Leiomyomata: Efficacy and Safety
Source: Cardiovasc Intervent Radiol. 2018 Oct 4;42(3):371–80. doi: 10.1007/s00270-018-2081-2 (PMC6373190; doi:10.1007/s00270-018-2081-2)

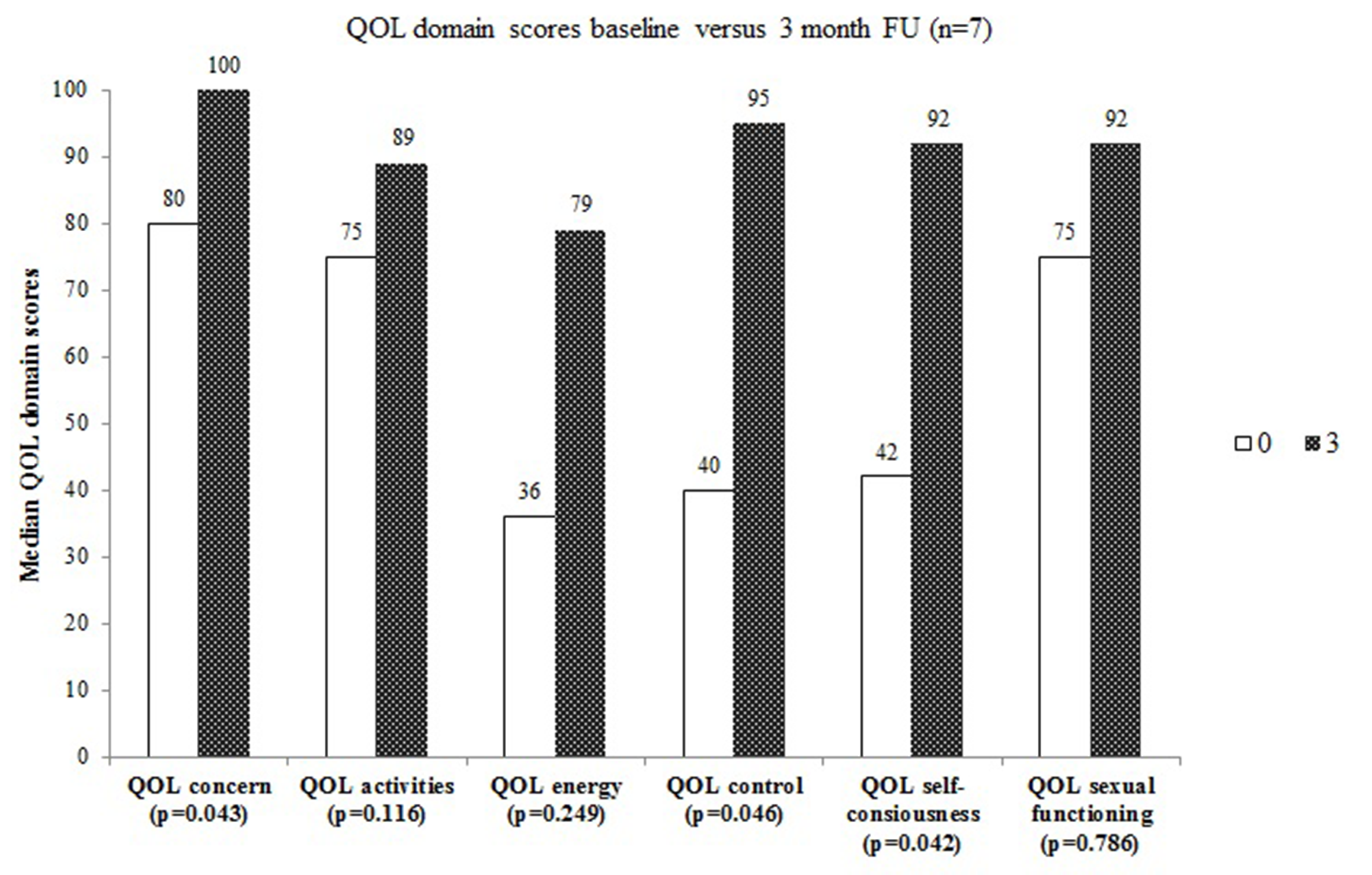

Supplement: Supplementary file 1 — Median quality of life subdomain scores at baseline and 3-month follow-up (TIFF 10035 kb) [file 270_2018_2081_MOESM1_ESM.tif]
